# Long-Term Spatiotemporal Reconfiguration of Neuronal Activity Revealed by Voltage-Sensitive Dye Imaging in the Cerebellar Granular Layer

**DOI:** 10.1155/2015/284986

**Published:** 2015-07-29

**Authors:** Daniela Gandolfi, Jonathan Mapelli, Egidio D'Angelo

**Affiliations:** ^1^Dipartimento di Scienze del Sistema Nervoso e del Comportamento, Università di Pavia, 27100 Pavia, Italy; ^2^Dipartimento di Scienze Biomediche, Metaboliche e Neuroscienze, Università di Modena e Reggio Emilia, 41125 Modena, Italy; ^3^Brain Connectivity Center, C. Mondino National Neurological Institute, 27100 Pavia, Italy

## Abstract

Understanding the spatiotemporal organization of long-term synaptic plasticity in neuronal networks demands techniques capable of monitoring changes in synaptic responsiveness over extended multineuronal structures. Among these techniques, voltage-sensitive dye imaging (VSD imaging) is of particular interest due to its good spatial resolution. However, improvements of the technique are needed in order to overcome limits imposed by its low signal-to-noise ratio. Here, we show that VSD imaging can detect long-term potentiation (LTP) and long-term depression (LTD) in acute cerebellar slices. Combined VSD imaging and patch-clamp recordings revealed that the most excited regions were predominantly associated with granule cells (GrCs) generating EPSP-spike complexes, while poorly responding regions were associated with GrCs generating EPSPs only. The correspondence with cellular changes occurring during LTP and LTD was highlighted by a vector representation obtained by combining amplitude with time-to-peak of VSD signals. This showed that LTP occurred in the most excited regions lying in the core of activated areas and increased the number of EPSP-spike complexes, while LTD occurred in the less excited regions lying in the surround. VSD imaging appears to be an efficient tool for investigating how synaptic plasticity contributes to the reorganization of multineuronal activity in neuronal circuits.

## 1. Introduction

Long-term synaptic plasticity is thought to represent the cellular basis of learning and memory in brain circuits [1]. Since its discovery in the early 1970s, various mechanisms and sites of expression have been described in detail [2]. However, the spatiotemporal distribution of synaptic plasticity has been poorly investigated, despite the fact that the reorganization of synaptic weights following long-term potentiation (LTP) and long-term depression (LTD) is fundamental for tuning signal transfer and for regulating homeostatic processes in distributed networks [3, 4]. Investigation of the spatiotemporal organization of synaptic plasticity demands the use of specific techniques making it possible to monitor the ensemble network activity.

While electrophysiological methods can provide relevant information on mechanisms and patterns of neuronal activity changes, they have been found to be of little value for describing the principles of organization of synaptic plasticity in neuronal networks [5]. An alternative strategy is to use optical methods to provide information on the spatiotemporal organization of neuronal activity [6–10]. In particular, VSD imaging [11], by correlating fluorescence variations with membrane potential changes [12, 13], allows the activity of neuronal circuits to be monitored both* in vitro* [5, 14, 15] and* in vivo* [16, 17]. VSD imaging is therefore a good candidate tool for mapping the spatiotemporal reorganization of neuronal circuit activity induced by synaptic plasticity.

Various forms of synaptic plasticity have been discovered in the cerebellar cortex [18]. In particular, theta burst stimulation (TBS) delivered to mossy fibers can differentially induce LTP or LTD in cerebellar granule cells (GrCs), depending on the postsynaptic membrane potential [19] and intracellular calcium level [7]. Furthermore, LTP and LTD are nonuniformly distributed according to lateral inhibition exerted by Golgi cells [20]. In this work, VSD imaging and patch-clamp recordings in acute cerebellar slices were used to detect LTP and LTD in the granular layer. VSD imaging could indeed detect LTP and LTD, providing a mechanistic correlation of signal changes with the underlying modifications in neuronal activity.

## 2. Materials and Methods

Experiments were performed using Sprague Dawley rats at postnatal day P17-P24 (internal breeding, Charles River (Calco, Lecco, Italy)). All experiments were conducted in accordance with the guidelines contained in the European Community Council Directive 86/609/EEC on the ethical use of animals.

### 2.1. Cerebellar Slices

Parasagittal cerebellar slices were obtained as previously described [5, 15, 21]. Briefly, rats were anesthetized with isoflurane (Sigma-Aldrich, Saint Louis, MO, USA) and decapitated. The cerebellum was removed and the vermis was isolated and fixed on a vibroslicer stage (VT1000S, Leica Microsystems, Nussloch, Germany) with cyanoacrylic glue. Acute 200 *µ*m thick slices were cut in cold cutting solution containing (in mM) 130 K-gluconate, 15 KCl, 0.2 EGTA, 20 HEPES, 10 glucose, and pH adjusted to 7.4 with NaOH. Slices were incubated at 32°C for at least 1 hour before recordings in oxygenated extracellular Krebs solution containing (in mM) 120 NaCl, 2 KCl, 1.2 MgSO_4_, 26 NaHCO_3_, 1.2 KH_2_PO_4_, 2 CaCl_2_, and 11 glucose (pH 7.4 when equilibrated with 95% O_2_ and 5% CO_2_). All drugs were obtained from Tocris Bioscience (Bristol, UK).

### 2.2. Patch-Clamp Recordings

Whole-cell recordings from GrCs were obtained by means of the patch-clamp technique [5, 21, 22] using a Multiclamp 700B amplifier (Molecular Devices, Union City, CA, USA) (−3 dB; cut-off frequency = 2 kHz). Recordings were digitized at 20 kHz using pClamp 10 (Molecular Devices) and a Digidata 1440A A/D converter (Molecular Devices). Patch pipettes were filled with the following solution (in mM): 126 K-gluconate, 8 NaCl, 15 glucose, 5 HEPES, 1 MgSO_4_, 0.1 BAPTA-4K, 0.05 BAPTA-Ca^2+^, 3 ATP, 100 *µ*M GTP, and pH adjusted to 7.2 with KOH. This solution maintained resting free Ca^2+^ concentration at 100 nM and pipettes had a resistance of 7–10 M*Ω* before seal formation.

Excitatory mossy fibers were stimulated by positioning a bipolar tungsten electrode (Clark Instruments, Pangbourne, UK) over the mossy fiber bundle (stimulation intensity: ± 5–15 V; 100 *μ*s, via a stimulation unit). Excitatory synaptic activity was generated starting from a membrane potential of between −55 and −65 mV (mean −59.3 ± 1.3,  *n* = 10). Current relaxation induced by a 10 mV step from the holding potential of −70 mV was analyzed to ensure stability of recordings. According to previous reports [21, 23, 24], transients were reliably fitted with a monoexponential function yielding membrane capacitance of 2.3 ± 0.3 pF, input resistance of 1.7 ± 0.2 G*Ω*, and series resistance of 18.5 ± 0.6 M*Ω* (*n* = 10). Spike probability was calculated as the number of times a GrC elicited at least one spike in response to a single mossy fiber stimulus over 10 repetitions.

### 2.3. VSD Imaging

VSD imaging was obtained as reported in [15, 25, 26]. Briefly, slices were incubated for 30 min in oxygenated Krebs solution added with 3% Di-4-ANEPPS stock solution mixed with 50% fetal bovine serum (Life Technologies, Carlsbad, CA, USA). The dye (Di-4-ANEPPS, Molecular Probes) was dissolved and stocked in Krebs with 50% ethanol (Sigma-Aldrich, Saint Louis, MO, USA) and 5% Cremophor EL (a castor oil derivative (Sigma-Aldrich, Saint Louis, MO, USA)). Slices were then transferred to a recording chamber and perfused at 1.5 ml min^−1^ with oxygenated Krebs solution maintained at 32°C by a thermostatic controller (Thermostat HC2, Multichannel system, GmbH, Reutlingen, Germany). Slices were immobilized with a nylon mesh attached to a platinum *Ω*-wire.

The recording chamber was installed on the stage of an upright microscope (BX51WI, Olympus, Europa GmbH, Hamburg, Germany) equipped with a 20x objective (XLUM Plan FL 0.95 NA). The light generated by a halogen lamp (150 W, MHF-G150LR, MORITEX Corp., Tokyo, Japan) was controlled by an electronic shutter (model0, Copal Co., Tokyo, Japan) and then passed through an excitation filter (*λ* = 530 ± 10 nm) projected onto a dichroic mirror (*λ* = 565 nm) and reflected toward the objective lens to illuminate the specimen. Fluorescence generated by the tissue was transmitted through an emission filter (*λ* > 590 nm) to the CCD camera (MICAM Ultima, Scimedia, Brainvision, Tokyo, Japan). The whole imaging system was connected through an I/O interface (Brainvision) to a PC controlling illumination, stimulation, and data acquisition. With this configuration, the final pixel size was 5 *μ*m. Full-frame image acquisition was performed at 1 kHz.

**Figure 1:**
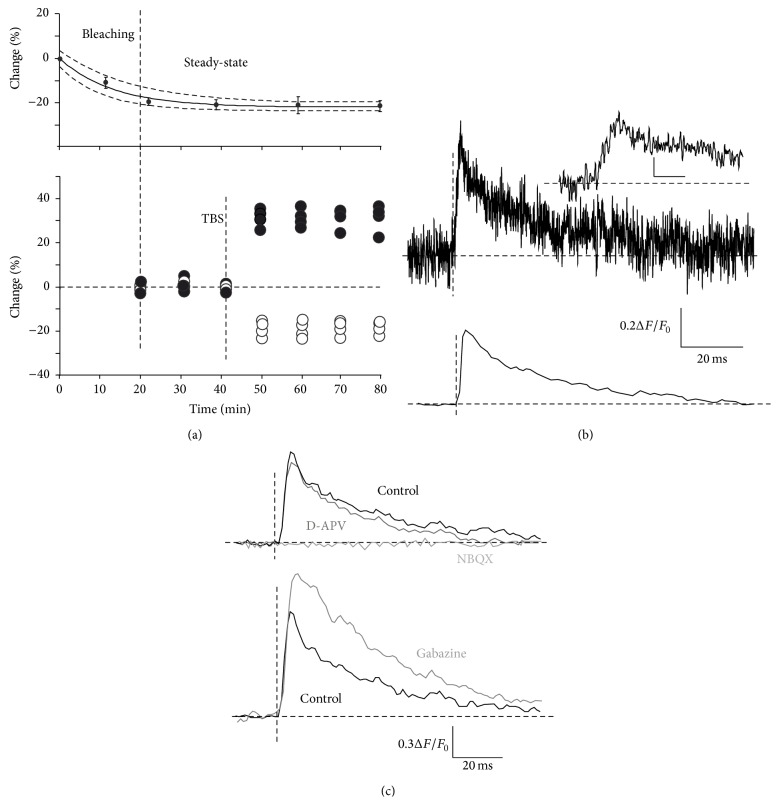
Properties of VSD imaging recordings. (a)* Top*. Time course of the peak amplitude of VSD signals averaged over five experiments in which TBS was not delivered. The signals tended to decrease exponentially in the first minutes of the acquisition to reach a steady-state level. The fitting function is represented over the experimental data, while dashed lines represent the fitting functions obtained by using the maximum deviations of the experimental data.* Bottom*. Time course of five regions of interest taken from a single experiment showing persistent positive or negative variations following TBS. Note that the control period used to calculate changes in VSD peak amplitude was taken in the steady-state region. (b)* Top*. VSD signal taken from granular layer and obtained in response to a single stimulus delivered to the mf. The trace was sampled at 2 kHz without averaging or filtering. The inset shows the initial phase of the VSD response (scale bar 10 ms and 0.1 Δ*F*/*F*_0_).* Bottom*. The trace shows the average of 10 repetitions sampled at 1 kHZ. (c)* Top*. VSD signals obtained in control solution (black trace) and in response to the application of D-APV (dark gray) and NBQX (light gray).* Bottom*. VSD signals obtained in control solution (black) and in the presence of 10 *µ*M gabazine.

Voltage-sensitive dyes are molecules intercalating into the outer leaflets of the membrane and undergoing changes in fluorescence spectra upon changes in the surrounding electric fields [12, 13]. Given these premises, two main limitations need to be carefully considered when performing VSD imaging recordings: photobleaching and phototoxicity of the dye. We experimentally estimated a long-term bleaching function by continuously monitoring the peak of response for one and a half hours (Figure 1(a) top panel). Experimental data were then fitted with an exponential decay function, **f**_bleach_ = *y*_0_ + *Ae* ^−*t*/*τ*^  (*y*_0_ = 0.62 ± 0.06; *A* = 0.35 ± 0.05; 1/*τ* = 0.018), and the standard deviations of the fitting parameters were used to generate an interval of confidence for signal decay. Signal intensity decreased by about 20% after 20 minutes before reaching a steady-state level. During synaptic plasticity recordings, the control period was calculated 20 minutes after the first stimulation (Figure 1(a), bottom panel). Moreover, as can be seen from Figure 1(b), which shows a comparison between a single acquisition at 2 kHz sampling frequency (Figure 1(b), top) and an average of 10 sweeps at 1 kHz sampling frequency (Figure 1(b), bottom), the signals remained stable without showing a significant decrease. It was thus not necessary to apply corrections for short-term photobleaching.

The recording protocol was organized as reported elsewhere [5, 15, 25, 26]. Briefly, each acquisition lasted for 500 ms and the stimulation protocol was triggered 50 ms after the shutter opening. Ten sweeps were repeated and averaged at the repetition frequency of 0.1 Hz to increase the signal-to-noise ratio (S/N). Given maximal Δ*F*/*F*_0_ ≈ 1% and noise SEM ≈ ±0.1% (*n* = 8 slices), the S/N was about 10 : 1 which ensured reliable peak amplitude measurement. The time courses of synaptic plasticity were generated by acquiring an average of 10 sweeps every 10 minutes to avoid photobleaching of the fluorescent molecules due to light overexposure. Data were acquired and displayed by Brainvision software. Signals were analyzed off-line using custom-written routines in MATLAB (Mathworks, Natick, USA). The granular layer point spread function [27] corresponded to that previously evaluated in the same experimental conditions [25].

Voltage-sensitive dye (VSD) responses were modulated by the activity of excitatory glutamatergic and inhibitory GABAergic receptors (Figure 1(c); see [25, 26]). Application of the NMDA_R_ blocker D-APV (25 *µ*M) reduced the peak amplitude (−17.3 ± 3.1%  *n* = 4,  *p* < 0.01, Figure 1(c), dark gray trace) as well as the late phase of the response (measured 50 ms after the stimulus onset; −31.8 ± 6.4%  *n* = 4, *p* < 0.01). The subsequent application of the AMPA_R_-selective blocker NBQX (10 *µ*M) completely abolished VSD responses (Figure 1(c), light gray trace). Finally, the application of the GABA_A_ receptor blocker gabazine (10 *µ*M) markedly increased the peak amplitude (+34.5 ± 5.8%  *n* = 4,  *p* < 0.01, Figure 1(c), bottom) and late phase response (+39 ± 6.1%,  *n* = 4, *p* < 0.01, Figure 1(c), bottom).

Synaptic plasticity was induced by eight bursts of 10 pulses at 100 Hz, which were repeated every 250 ms (TBS).

### 2.4. Data Analysis

In a defined time window, an automatic procedure detected the average fluorescence signal before stimulus (*F*_0_) and the local maximum after stimulus (*F*). The automatic procedure calculated the difference between *F* and *F*_0_, giving the relative peak amplitude (Δ*F*). This made it possible to measure the following parameters: peak amplitude and time taken to reach the response peak from stimulus onset (time-to-peak). The peak amplitude and time-to-peak of VSD signals collected in each pixel were normalized to maximum values among all the pixels in each experiment. Activation vectors were generated by representing, on Cartesian coordinates, normalized peak amplitude (*y*) and time-to-peak (*x*). Vector slopes were calculated as the ratio between the relative fluorescence change (Δ*F*/*F*_0_) and the time-to-peak of VSD signals. The initial slope was calculated as the ratio between Δ*F*/*F*_0_ measured in the first 5 ms following the beginning of VSD response divided by 5 ms. Activation maps were generated by assigning to each pixel of the matrix the corresponding value of peak amplitude, time-to-peak, or vector slope obtained from VSD signals. Pseudocolors and spatial interpolation were used to improve graphical reconstruction. VSD signals were classified into distinct groups using a principal component analysis (PCA, MATLAB, Mathworks, Natick, USA) algorithm. PCA made it possible to separate the activation vectors into two physiologically relevant groups. Slopes were classified as “large” or “small” (named, resp., group 1 and group 2 in the text) by applying PCA to each recording.

Average spots of activation were generated by taking 100 × 100 *μ*m^2^ areas centered on a local peak amplitude maximum, aligned along the mossy fiber axis, and averaged with regions taken from different experiments (see [19], *n* = 7056 pixels, *n* = 8 slices). Spot diameters were estimated by fitting both axes (4 pixel-wide stripes) of the activated areas with sigmoidal functions [*y*(*x*) = *A*_0_ + *A*_1_/(1 + exp(*x*_half_ − *x*)/rate). The average activation radius was estimated as the half-amplitude width (*x*_half_), while the profile slope was estimated as the function rate. The average activation spot diameter was 54.5 ± 3.7 (*n* = 7056 pixels, *n* = 8 slices), which is perfectly in line with recently reported values (see [10]).

The intensity and sign of synaptic plasticity were estimated by calculating, through a custom-written procedure (MATLAB, Mathworks, Natick, USA), the difference between average values in the control period and after TBS (peak amplitude, time-to-peak, and vector slope). Only variations persisting until the end of recordings were considered as LTP or LTD. Moreover, stochastic noise introduced by automatic detection procedures was reduced by discarding pixels showing variations during the control period that were ±*σ* (standard deviation) larger than the average of the control period. To prevent ambiguity in the interpretation of small changes, variations < 10% were not considered as LTP or LTD.

Data are reported as mean values ± standard error of the mean (SEM) and statistical comparisons were performed using paired Student's *t*-test unless otherwise stated.

## 3. Results

### 3.1. Clustering of Two Types of VSD Signals in the Cerebellar Granular Layer

**Figure 2:**
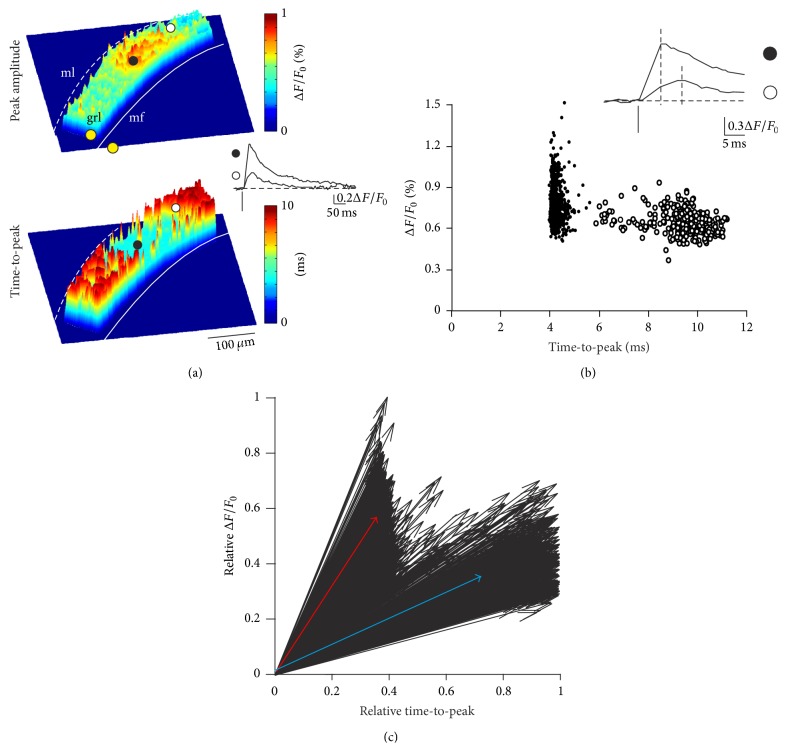
Vector representation identifies two populations of VSD signals in the cerebellar granular layer. VSD imaging of granular layer activity in a rat parasagittal slice. In this and in the following figures mf = mossy fibers (continuous white line); grl = granular layer; ml = molecular layer; dashed white lines = Purkinje cell layer; yellow dots = stimulation electrodes. (a)* Top*. Activation map of the granular layer in response to a single pulse delivered to a mossy fiber bundle, in which pseudocolors represent response peak amplitudes.* Bottom*. Time-to-peak map for the same signals reported above (top). Traces illustrate time courses obtained from a region (filled circle) in which responses show large peak amplitude and short delay and a region (empty circle) with responses showing small peak amplitude and long delay. (b) The plot shows the Cartesian relationship between time-to-peak andpeak amplitude for the same experiment shown in (a). PCA analysis made it possible to distinguish between two groups of responses (filled and empty circles), which corresponded to the categories of signals observed in (a) and shown in the* inset*. (c) Signals shown in (b) were normalized for both peak amplitude and time-to-peak and represented as vectors. The thick blue vector results from averaging the empty circles in (b), while the thick red vector results from averaging the filled circles in (b).

Stimulation of the mossy fiber bundle in acute cerebellar slices elicited VSD responses in the granular layer. In line with previous observations [15, 25, 26], VSD responses were organized in spots (Figure 2(a)) with intensity degrading around a central, highly responsive area (cf. [25]; an example of spot reconstruction is shown in Figure 4(d); see below). Once averaged (see Section 2), spot responses revealed a center-surround organization similar to that reconstructed using multielectrode array recordings [19]. The VSD activity core had a diameter of 54.5 ± 3.7 *μ*m, a result closely matching the size measured by using two-photon imaging with single cell resolution (58 *μ*m; see [10]).

The VSD signals showed variable kinetics that were identified by measuring peak amplitude and time-to-peak in every pixel and then reconstructing maps to yield the spatial arrangement of granular layer activity (Figure 2(a)). Signals in the core of active areas were characterized by shorter time-to-peak and larger peak amplitude than those in the surround (0.9 ± 0.1%  Δ*F*/*F*_0_ and time-to-peak 5.8 ± 0.3 ms versus 0.65 ± 0.2%  Δ*F*/*F*_0_ and time-to-peak 10.4 ± 0.4 ms; *n* = 21176 pixels, 8 slices; Figure 2(a)).

The plot of peak amplitude* versus* time-to-peak of the VSD signals measured in individual pixels (Figure 2(b)) revealed two separate clusters of data points, one characterized by large peak amplitude and short time-to-peak and the other characterized by small peak amplitude and long time-to-peak. Peak amplitude and time-to-peak were normalized to maximum values and their combination resulted in activation vectors, which also formed two distinct clusters (Figure 2(c)). Principal component analysis (see Section 2) confirmed the presence of two distinct groups (average slopes 1.33 ± 0.11 versus 0.62 ± 0.13; *n* = 21176 pixels, 8 slices; Figure 2(c)). A PCA was performed and two groups of vectors were identified for each recording and classified as “large” slopes (group 1) and “small” slopes (group 2).

The analysis of the two groups revealed that they originated from different responsive areas. The majority of pixels belonging to group 1 were in a core of activation (75.5 ± 4.6% based on peak amplitude; 73.1 ± 5.2% based on time-to-peak; *n* = 8 slices, *n* = 7893 pixels), while most of group 2 pixels were in the surround (80.1 ± 2.5% based on peak amplitude; 76.9 ± 3.1% based on time-to-peak; *n* = 8 slices, *n* = 13283 pixels).

### 3.2. VSD Signals Correlate with the Nature of the Underlying Neuronal Responses

**Figure 3:**
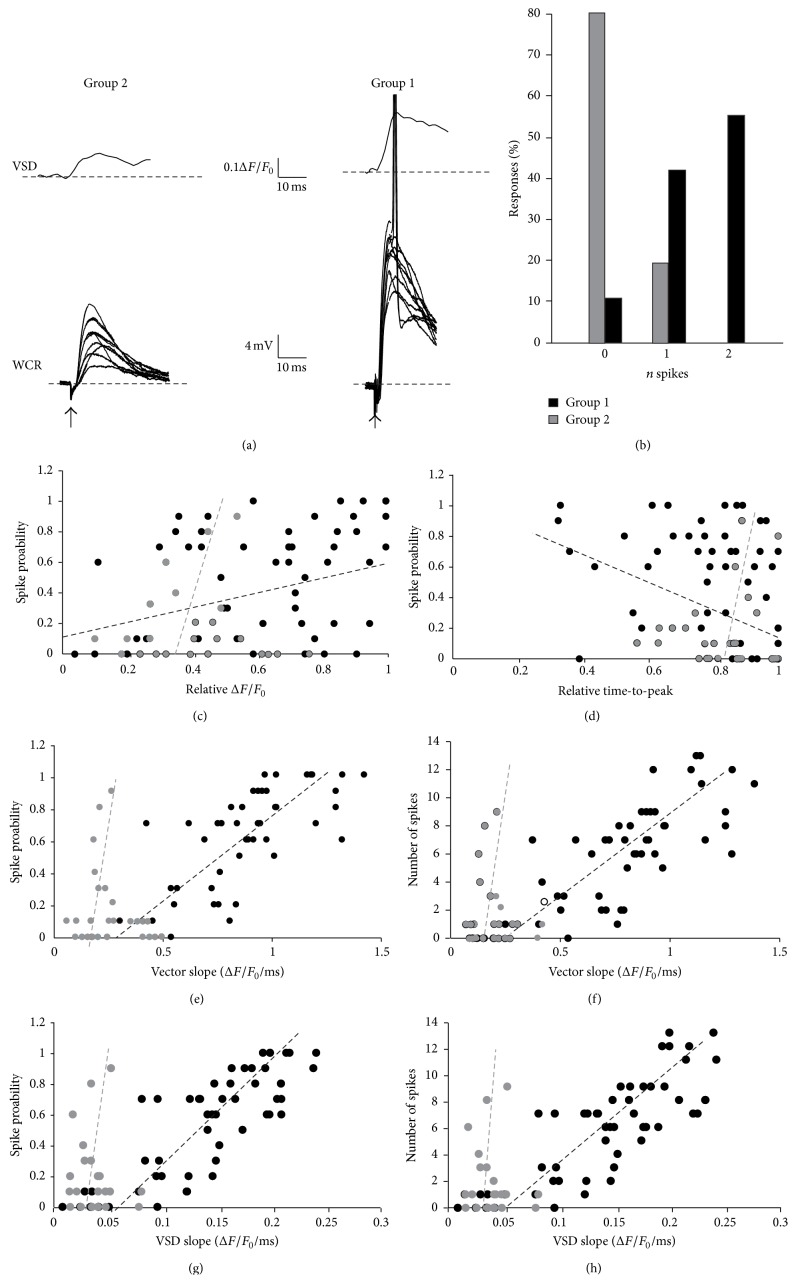
Correlation of VSD signals and intracellular granule cell activity. (a) VSD signals with a* small slope* or* large slope* were obtained from ROIs corresponding to the somata of GrCs recorded intracellularly and compared to electrical traces. The recording from a GrC in a low-responding region shows a prevalence of EPSPs (10 consecutive sweeps are superimposed). The recording from a GrC in a high-responding region shows larger EPSPs and EPSP-spike complexes (10 consecutive sweeps). (b) The histogram shows the number of spikes emitted by GrCs during 10 consecutive sweeps in ROIs belonging to group 1 or group 2. (c) Correlation between normalized peak amplitude and the probability of eliciting at least one action potential per GrC during 10 consecutive sweeps. Linear regression lines are shown for both clusters of VSD signals emerging from PCA (dashed black line,* group 1* ROIs, *R* ^2^ = 0.2, Fisher's *F*-test *p* = 0.04; dashed gray line,* group 2* ROIs, *R* ^2^ = 0.37, Fisher's *F*-test *p* = 0.02). (d) Correlation between normalized time-to-peak and the probability of eliciting at least one action potential per GrC during 10 consecutive sweeps. Linear regressions are shown for both clusters of VSD signals emerging from PCA (dashed black line,* group 1* ROIs, *R* ^2^ = 0.15, Fisher's *F*-test *p* = 0.06; dashed gray line,* group 2* ROIs, *R* ^2^ = 0.16, Fisher's *F*-test *p* = 0.03). (e) Correlation between the vector slope of VSD signals and the probability of eliciting at least one action potential per GrC. Linear regressions are shown for both clusters of VSD signals emerging from PCA (dashed black line,* group 1* ROIs, *R* ^2^ = 0.85, Fisher's *F*-test *p* = 10^−14^; dashed gray line,* group 2* ROIs, *R* ^2^ = 0.89, Fisher's *F*-test *p* = 10^−15^). (f) Correlation between the slope of the VSD signals and the total number of emitted spikes per GrC. Linear regressions are shown for both clusters of VSD signals emerging from PCA (dashed black line,* group 1* ROIs, *R* ^2^ = 0.82, Fisher's *F*-test *p* = 10^−11^; dashed gray line,* group 2* ROIs, *R* ^2^ = 0.84, Fisher's *F*-test *p* = 10^−13^). (g) Correlation between the initial slope of the VSD signals and the probability of eliciting at least one action potential per GrC. Linear regressions are shown for both groups of VSD signals emerging from PCA (dashed black line,* group 1 *ROIs, *R* ^2^ = 0.91, Fisher's *F*-test *p* = 10^−16^; dashed gray line,* group 2 *ROIs, *R* ^2^ = 0.78, Fisher's *F*-test *p* = 10^−9^). (h) Correlation between the initial slope of the VSD signals and the total number of emitted spikes per GrC. Linear regressions are shown for both groups of VSD signals emerging from PCA (dashed black line,* group 1 *ROIs, *R* ^2^ = 0.92, Fisher's *F*-test *p* = 10^−14^; dashed gray line,* group 2* ROIs, *R* ^2^ = 0.8, Fisher's *F*-test *p* = 10^−7^).

Combined VSD and whole-cell current-clamp recordings from GrCs (Figures 3(a) and 3(b)) were performed to investigate the cellular origin of the differences observed in VSD responses. Electrical activity elicited by mossy fiber stimulation was compared with VSD signals taken from regions of interest (ROIs) corresponding to the somata of recorded GrCs (see [25, 26]). In response to a single mossy fiber stimulus, GrCs generated either EPSPs or EPSPs-spike complexes, probably reflecting different balances of synaptic inhibition and excitation (Figures 3(a) and 3(b); see [21, 28]). ROIs showing small responses and belonging to group 2 were associated with GrCs responding with a prevalence of EPSPs (Figure 3(a), *n* = 5 cells). Conversely, ROIs showing large responses and belonging to group 1 were more likely to be associated with GrCs responding with EPSP-spike complexes (Figure 3(b), *n* = 8 cells). VSD signals were also correlated with the number of emitted spikes. Recordings from GrCs located in low-responding ROIs showed a tendency to generate EPSPs or just rare action potentials, whereas GrCs recorded in high-responding regions reliably generated 1-2 action potentials per stimulus (Figure 3(b)).

The probability of the GrCs generating spikes was correlated with the slope of the corresponding VSD signals (see Section 2 for details). Although the correlation was modest when either single amplitude (Figure 3(c)) or time-to-peak was used (Figure 3(d)), a much more robust relationship emerged from the correlation between vector slopes and spike probability or total number of spikes. In this case, group 1 vectors showed *R* ^2^ = 0.85 (Fisher's *F*-test *p* < 10^−14^; *n* = 8 cells, Figure 3(e) black circles) and group 2 vectors showed *R* ^2^ = 0.79 (Fisher's *F*-test *p* < 10^−8^; *n* = 5 cells, Figure 3(e) gray circles) when correlated with spike probability, while group 1 vectors showed *R* ^2^ = 0.82 (Fisher's *F*-test *p* < 10^−11^; *n* = 8 cells, Figure 3(f) black circles) and group 2 showed *R* ^2^ = 0.75 (Fisher's *F*-test *p* < 10^−8^; *n* = 5 cells, Figure 3(f) gray circles). Finally, a robust correlation also emerged when spike probability and the total number of emitted spikes were correlated with the initial slope of the VSD signal (Figures 3(g) and 3(h); see Section 2) in order to limit noise deriving from postsynaptic spikes (see Figures 3(g) and 3(h) for details).

These data indicated that activation of GrCs was reflected in VSD signal kinetics, and by using vector representation two classes of VSD responses could be identified.

### 3.3. VSD Imaging Can Reveal LTP and LTD

The application of TBS to mossy fibers is known to induce LTP or LTD at the cerebellar mossy fiber-GrC synapse depending on the local excitatory/inhibitory balance [19]. We investigated whether VSD imaging could be used to map the organization of synaptic plasticity in neuronal circuits of the granular layer at high spatial resolution.

**Figure 4:**
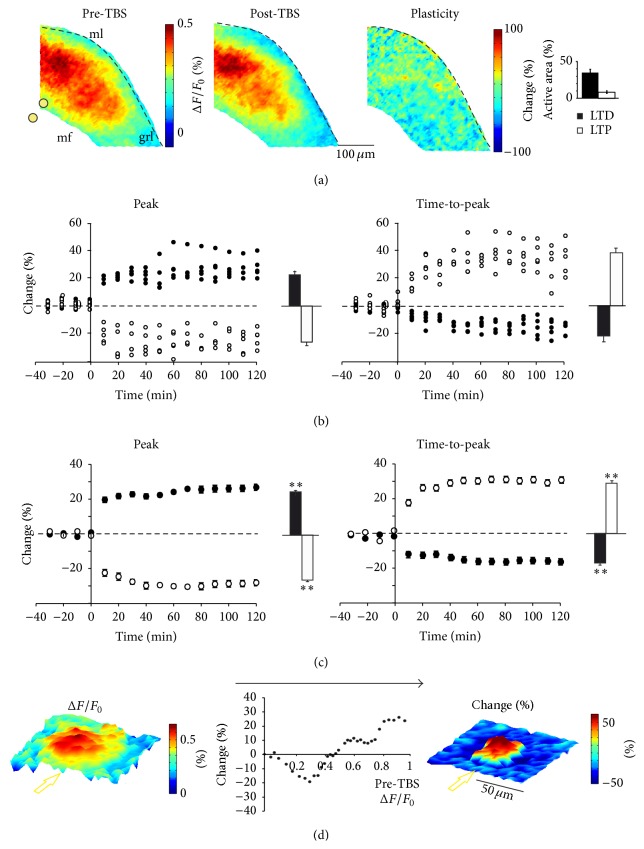
Properties of LTP and LTD. (a) Activation maps of the granular layer in response to a single pulse delivered to a mossy fiber bundle before (*left*) and after (*middle*) TBS. The distribution and intensity of the changes induced by TBS are shown in the subtraction map (*right*; see Section 2). The histogram shows the percentages of active pixels displaying LTP or LTD. (b) Time courses of VSD signal changes in five pixels showing LTP and five pixels showing LTD taken from the maps shown in (a). Pixels demonstrate stable responses in the control period and persistent variations of peak amplitude (left) and time-to-peak (right) after TBS. Note the inverse sign of the changes in the time course of peak amplitude and time-to-peak. The histograms show average changes in the 30–60 minutes after TBS compared to the control period. (c) Average time courses of all pixels showing LTP (*n* = 7 slices, *n* = 2603 pixels) and LTD (*n* = 8 slices, *n* = 10116 pixels) both for peak amplitude and time-to-peak. Histograms show average changes in the period 30–60 minutes after TBS compared to the control period. (d) Correspondence between theaverage center-surround map of VSD activation (*left*; *n* = 8 slices and 7056 pixels) and plasticity (*right*; *n* = 7 slices and 6174 pixels) (the maps were generated by centering over pixels showing maximal response or maximal LTP and realigning the slices along the mossy fiber bundle indicated by the yellow arrow; see Section 2). The plot (*center*) shows the relationship between excitation measured as initial VSD amplitude and relative changes following TBS (the data were obtained only from pixels displaying significant and persistent variations).

Plasticity maps were generated by subtracting average VSD maps obtained in control period (average of 30 minutes before TBS) from maps obtained after TBS (average 30–60 minutes after TBS) (Figure 4(a)). TBS induced either LTP or LTD with time courses similar to those observed with other experimental techniques (see [7, 19, 22, 29]) (Figure 4(b)). LTP appeared as a persistent increase in the VSD signal peak amplitude and was associated with a persistent decrease in the VSD signal time-to-peak (Figure 4(b)). Conversely, LTD appeared as a persistent decrease in the VSD signal peak amplitude and was associated with a persistent increase in VSD signal time-to-peak (Figure 3(b)). LTP and LTD coexisted in seven out of the eight slices tested, while LTD alone was observed in the remaining slice. In all cases, the number of LTD pixels was greater than the number of LTP pixels (35.3 ± 4.7% versus 8.1 ± 2.4% of active areas *p* < 0.01; the remaining pixels did not show stable changes; Figure 3(a)). On averaging VSD signals obtained from pixels showing persistent changes (>10% over 45 minutes; see Section 2), LTP was found to show a peak amplitude increase of +25.9 ± 0.8% and a time-to-peak decrease of −18.4 ± 0.6% (*n* = 2603 pixels, *n* = 7 slices *p* < 0.001; Figure 4(c)), whereas LTD showed a peak amplitude decrease of −26.9 ± 0.7% and a time-to-peak increase of +37.9 ± 0.5% (LTD, *n* = 10116 pixels, *n* = 8 slices *p* < 0.001; Figure 4(c)). These data are consistent with results obtained with different experimental methods, confirming the reliability and stability of VSD data recordings [7, 19, 22, 29].

The spatial organization of LTP and LTD could be visualized using a reconstruction of the average center-surround before and after plasticity, which was carried out on all the eight slices covered in this study (Figure 4(d)). Interestingly, the core, which generated stronger responses than the surround, tended to express LTP rather than LTD. Moreover, the relationship between basal excitation and plasticity taken from all pixels revealed a sigmoidal plot reminiscent of Bienenstock et al.'s [30] plasticity rule reported previously for granular layer synapses [7, 19].

### 3.4. Spatial Evolution of Local Responses during Long-Term Synaptic Plasticity

**Figure 5:**
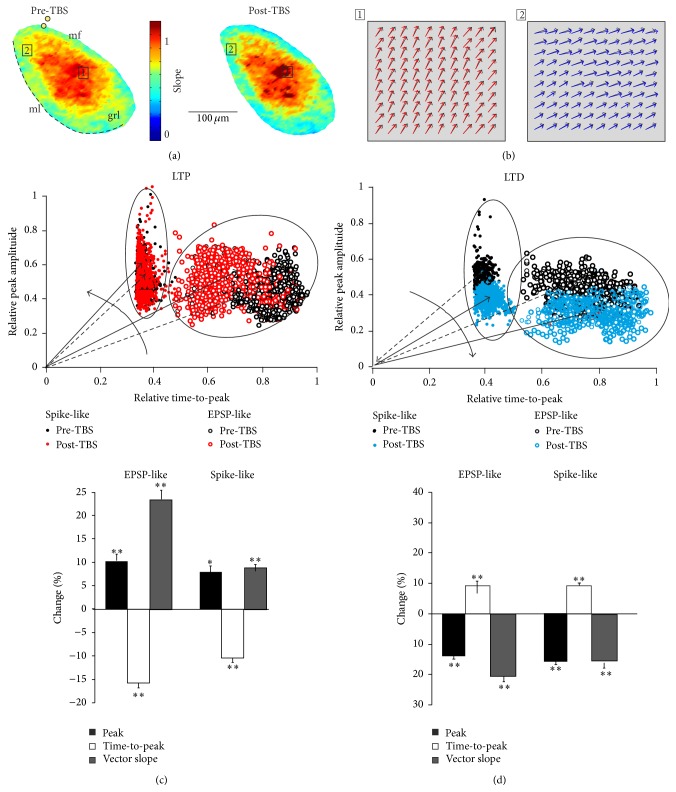
Activation vectors allow mapping LTP and LTD. (a) Vector maps of the granular layer before (left) and after TBS (right). The colors represent the vector slopes. (b) Individual vectors of pixels showing LTP in a high-responding region (square 1) were obtained before and after TBS (black and red vectors, resp.). Similarly, individual vectors of pixels showing LTD in a low-responding region (square 2) were obtained before and after TBS (black and blue vectors, resp.). (c)* Up*. Activation vectors undergoing LTP are divided into two clusters based on PCA (spike-like, filled circles; EPSP-like, empty circles). LTP increases the average vector slope (from dashed to filled arrows).* Down*. Activation vectors undergoing LTD are divided into clusters based on PCA (spike-like, filled circles; EPSP-like, empty circles). LTD decreases the average vector slope (from dashed to filled arrows). (d) The histograms summarize the effects of LTP and LTD on peak amplitude, time-to-peak, and vector slopes in the EPSP-like and spike-like clusters (Student's *t*-test statistical significance: ^*∗*^ *p* < 0.05; ^*∗∗*^ *p* < 0.01).

Since changes in peak and time-to-peak of VSD signals were indicative of LTP and LTD, activation vectors were used to analyze the cellular correlates of modifications occurring after TBS. Vector maps were generated by assigning to every pixel the corresponding vector slope obtained from VSD signals (Figures 5(a) and 5(b)). The vector maps showed that, in response to TBS, vectors in regions displaying larger slopes and stronger excitation (Figure 5(a), square 1) tended to become steeper (Figure 5(b), left). Conversely, VSD responses exhibiting small slopes, deriving from poorly activated areas (Figure 5(a) square 2), became less steep (Figure 5(b), right).

**Figure 6:**
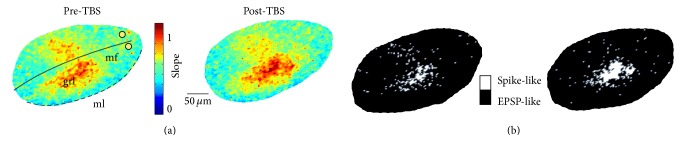
Spatial reconfiguration by LTP and LTD. (a) Vector maps of granular layer before (left) and after TBS (right). (b) The maps are represented by converting spike-like signals into white pixels and EPSP-like signals into black pixels. Plasticity increases the density and amount of spike-like responses in the core of the active area and filters out spike-like responses from the surround.

The impact of LTP and LTD on VSD signals was assessed by evaluating the vector slope increase (LTP effect, Figure 5(c)) and vector slope decrease (LTD effect, Figure 5(d)) on both EPSP-like and spike-like responses separately. LTP increased spike-like responses (vector slope +8.2 ± 1.7%,  *p* < 0.01; *n* = 7 slices, *n* = 1556 pixels), reflecting a peak amplitude increase (8.3 ± 1.3%, *p* < 0.05) and a time-to-peak decrease (−10.5 ± 0.9%, *p* < 0.01). LTP also increased EPSP-like responses (vector slope +23.9 ± 5.5%, *p* < 0.001; *n* = 7 slices, *n* = 1047 pixels) reflecting a peak amplitude increase (9.4 ± 1.9%, *p* < 0.001) and a time-to-peak decrease (−15.7 ± 2.1%, *p* < 0.001). LTD decreased spike-like responses (vector slope −16.5 ± 2.4%, *p* < 0.001; *n* = 8 slices, *n* = 2347 pixels), reflecting a peak amplitude decrease (−9.3 ± 2.1%  *p* < 0.001) and a time-to-peak increase (+9.7 ± 2%, *p* < 0.001). LTD also decreased EPSP-like responses (−19.7 ± 2.5%, *p* < 0.001; *n* = 8 slices, *n* = 7813 pixels), reflecting a peak amplitude decrease (−16.8 ± 1.9%, *p* < 0.001) and a time-to-peak increase (+9.8 ± 2.1%, *p* < 0.001). The majority of spike-like responses underwent LTP (65.8 ± 2.7%, *p* < 0.01; *n* = 7 slices *n* = 1556 pixels), while only a small amount of EPSP-like responses underwent LTP (13.8 ± 1.7%; *n* = 7 slices *n* = 1047 pixels; *p* < 0.001). Consistently, the density of EPSP-spike responses markedly increased in the core of the response but decreased in the surround (Figures 6(a) and 6(b)).

## 4. Discussion

The main observation from this study is that VSD imaging can be efficiently employed to detect activity changes caused by LTP and LTD in the cerebellar granular layer with* quasi-cellular* resolution. Distinct populations of signals with EPSP-like and spike-like properties showed differential localization, with spike-like responses being clustered in the core of the active area and EPSP-like responses in the periphery. LTP occurred 1.48 times more frequently in spike-like responses than in EPSP-like responses, while LTD occurred 3.3 times more frequently in EPSP-like responses than in spike-like responses, and as a whole LTD involved 3.9 times more pixels than LTP. Moreover, LTP was associated with decreased time-to-peak and LTD was associated with increased time-to-peak of the VSD signals. Therefore, VSD imaging could reveal the spatiotemporal reorganization of neuronal activity during LTP and LTD.

### 4.1. Quasi-Cellular Resolution and Stability of VSD Signals

A major limitation of VSD imaging recordings is the temporal resolution of the system. Although VSD molecules react rapidly (in a few microseconds) to membrane potential changes, increasing the acquisition rate lowers the S/N [31]. To follow the temporal evolution of fluorescence changes, it is therefore fundamental to achieve the optimal compromise between the acquisition frequency and the resulting S/N. The use of pixelated detectors, such as CMOS or CCD cameras, strongly limits the sampling rate because of the dramatic decrease in sensitivity below 1 ms of exposure time. This, in turn, makes it necessary to use averaging procedures, which lowers the possibility of discriminating events depending on stochastic mechanisms such as action potentials. The inferior limit of a recording system is determined by a combination of dye efficiency and detector sensitivity. VSD molecules shift their excitation and emission spectra by few nanometers upon large membrane potential variations. It would be therefore useful to excite with high-power monochromatic light in the steep tail of the spectrum in order to maximize Δ*F*/*F*_0_ by virtue of larger fluorescence variations [32]. However, this configuration often leads to strong photobleaching with a consequent dye phototoxicity that would be detrimental for long-term recordings. In our recordings, we did not observe photobleaching during single acquisitions (see Figure 1), which suggests that dye degradation and phototoxicity were negligible. However, a significant reduction in signal intensity was observed (Figure 1(b)) in the first minutes of recordings, which could be attributed to a slow decrease of dye sensitivity and dye washout in the early phase of the perfusion of fresh solution.

The final resolution of the VSD imaging system is also dependent on light scattering in the sample [33], which in cerebellar slices has been found to limit spatial resolution over scales compatible with one or two neuronal diameters (point spread function ~10 *μ*m) [25, 26]). Unfortunately, when using nonconfocal regimes, it is not possible to isolate the contribution of single neurons to the generation of VSD responses. Uncorrelated signals generated in multiple focal planes and from adjacent positions in the same plane increase the total noise of the pixelated detector. Thus, although the use of a monochromatic source would improve the efficiency of the dye, signal deconvolution algorithms would be necessary in order to attempt to discriminate the origin of the signals.

Although the combination of temporal and spatial resolution did not allow resolution of individual spikes in single GrCs, the combination of peak amplitude and time-to-peak measurements enhanced feature extraction from VSD signals. The vector representation permitted the categorization of neuronal responses into two fundamental groups: EPSP-like and spike-like. The close relationship between the initial slope of VSD signals, which should be less dependent on the presence of action potentials, and single-cell parameters supported the use of vector representation. This* quasi-cellular* resolution was confirmed by the correlation between vector representations of VSD signals and intracellular recordings of membrane potential [25, 26].

The possibility of discriminating between EPSP-like and spike-like responses, combined with the high stability of the recording system [15, 25, 26], made it possible to monitor long-term plasticity using VSD recordings and to correlate it with the underlying cellular processes.

### 4.2. Possible Sources of VSD Signal Contamination

The origin of VSD signals resides in the activity of granular layer neurons [25, 26], as confirmed by their dependence on postsynaptic receptor blockers (Figure 1(b)). However, glial cells, whose membranes are well stained by VSD molecules, can be a possible source of VSD signal contamination [34]. Nevertheless, synaptic activation evokes responses in astrocytes with kinetics that are much slower (tens or hundreds of milliseconds, [34]) than those in neurons (a few ms in GrC recordings [21]). It is therefore unlikely that glial cells contributed to generating the rapid time courses observed in VSD signals. A further source of contamination could be presynaptic depolarization propagating during synaptic stimulation. However, (i) mossy fibers are myelinated [35] and presynaptic depolarization is predominantly generated within presynaptic terminals in the glomeruli [29]; (ii) the density of GrC somata is estimated to be 4 × 10^6^/mm^3^, while that of glomeruli is 3 × 10^5^/mm^3^ [36], (iii) it has also been estimated that only 34% of the total glomerular volume is occupied by mossy fiber terminals [36]; (iv) the postsynaptic surface generating VSD signals is about 40 times larger than the presynaptic surface; (v) VSD signals were completely blocked in the presence of AMPA and NMDA receptor blockers.

These observations strongly suggest that VSD signals mainly originate from GrC depolarization occurring in response to mossy fiber stimulation rather than from action potentials originating in presynaptic terminals. The changes in VSD responses were thus likely related to persistent changes in GrC synaptic responsiveness elicited by the induction protocol, although a minor contribution coming from presynaptic mossy fiber terminals, glial cells, or other neuronal subtypes (e.g., Golgi cells) cannot be ruled out.

### 4.3. Long-Term Spatiotemporal Reconfiguration of Neuronal Activity

The effect of LTP on the VSD signals included a remarkable reduction of time-to-peak together with a peak amplitude increase, confirming that spike anticipation and spike synchronization are major effects of LTP in GrCs [22]. Conversely, the increased time-to-peak associated with LTD was modest compared to the peak amplitude decrease. This latter effect could be explained by the observation that LTD occurred mostly in responses that were already subthreshold, with the result that effects related to spike distribution and positioning could not be revealed. Consistent with the center-surround organization of granular layer responses to mossy fiber stimulation, previously reported [10, 19], LTP changes appeared clustered in the core and LTD changes in the periphery of the activated areas. This brought about a conversion of the core responses into EPSP-spike complexes and of the surround responses into EPSPs, generating a strong contrast enhancement that could be functional to raise the S/N along the transmitting pathway.

## 5. Conclusions

Despite intrinsic limitations due to light scattering, VSD imaging combined with vector signal analysis provided quasi-cellular resolution of the changes occurring in cerebellar microcircuit activity during long-term synaptic plasticity. Both EPSP-like and spike-like responses showed stable long-term plastic changes in the form of LTP or LTD, with a spatial distribution compatible with lateral inhibition generated by Golgi cells in the granular layer circuit. LTP changes reflected the anticipation and synchronization of spikes, while the opposite would occur with LTD [22, 37]. VSD imaging could therefore provide mechanistic insight into the changes caused by long-term synaptic plasticity in neuronal responsiveness supporting a primary role of the cerebellar granular layer in spatiotemporal reconfiguration of mossy fiber signals.
